# Multiscale Soft Surface Instabilities for Adhesion Enhancement

**DOI:** 10.3390/ma15030852

**Published:** 2022-01-23

**Authors:** Vaisakh Vilavinalthundil Mohanan, Ho Yi Lydia Mak, Nishan Gurung, Qin Xu

**Affiliations:** 1 Department of Physics, Faculty of Sciences, The Hong Kong University of Science and Technology, Clear Water Bay, Hong Kong SAR, China; vvm@connect.ust.hk (V.V.M.); H.Y.L.Mak@student.tudelft.nl (H.Y.L.M.); 2 Department of Geoscience and Remote Sensing, Faculty of Civil Engineering and Geosciences, Delft University of Technology, 2628 CN Delft, The Netherlands; 3 Department of Mathematics, Faculty of Sciences, The Hong Kong University of Science and Technology, Clear Water Bay, Hong Kong SAR, China; ngurung@connect.ust.hk

**Keywords:** instabilities, adhesion, soft gels, swelling

## Abstract

Soft polymeric gels are susceptible to buckling-induced instabilities due to their great compliance to surface deformations. The instability patterns at soft interfaces have great potential in engineering functional materials with unique surface properties. In this work, we systematically investigated how swelling-induced instability patterns effectively improved the adhesive properties of soft polydimethylsiloxane (PDMS) gels. We directly imaged the formations of the surface instability features during the relaxation process of a swollen gel substrate. The features were found to greatly increase the adhesion energy of soft gels across multiple length scales, and the adhesion enhancement was associated with the variations of contact lines both inside the contact region and along the contact periphery. We expect that these studies of instability patterns due to swelling will further benefit the design of functional interfaces in various engineering applications.

## 1. Introduction

Adhesive contacts of soft materials are appealing to scientists due to their essential roles in many engineering applications, such as 3D-printing architectures [1,2], flexible electronics [3,4], compliant robotics [5], microfluidic systems [6,7], bio-medical materials [8,9], etc. The low elastic moduli of these soft interfaces allow large surface deformations to sustain mechanical contacts. Depending on the contact geometries and local strain gradients, soft materials are susceptible to buckling-induced surface instabilities under compressions [10,11,12]. To quantitatively control the contact mechanics of soft polymeric gels, the understanding of how surface instability patterns affect soft adhesion is of crucial importance.

Considerable works have studied the rich mechanics of different instability patterns at soft interfaces, including buckling [13,14,15], wrinkles [15,16,17], creases [15,18], and folds [15,19,20]. A soft substrate covered with a stiff skin can easily generate wrinkles under uniaxial compressions. The characteristic wavelength of the patterns is determined by the difference in elastic moduli between the two layers and scales linearly with the skin thickness [21]. Wrinkles have been widely used to design functional soft interfaces [22,23,24,25]. For instance, soft polydimethylsiloxane (PDMS) gels treated with oxygen plasma can easily generate wrinkles under compressions, and their adhesion energy can be quantitatively adjusted by mechanical strains [26,27]. In addition, wrinkles were used to control the surface tribology of soft materials [28,29] and to design superhydrophobic structures [30,31].

Swelling of a soft polymer gel under confinement is another effective approach to generate surface instabilities [32,33,34]. Unlike the wrinkles in soft multilayer systems, the instability patterns induced by swelling are often referred to as creases [10,35,36]. While both wrinkles and creases result from free-energy minimization in buckling transitions, the growth mechanisms of the two instabilities are different [37,38]. Compared to wrinkles, crease formations involve substantial compressive strains, such that the patterns are often mechanically irreversible. Previous studies on creasing instabilities have focused on hydrogels. For instance, as a crosslinked hydrogel attached to a stiff substrate was fully immersed in an ionized water solution, restricted swelling along the vertical direction resulted in various crease-like patterns on gel surfaces [34]. Further investigations showed that creasing instabilities can be driven by external stimuli, such as temperature [39], light [40], and electric fields [41]. Due to the similar compositions between hydrogels and many biological materials, creases in hydrogels have been considered as model systems to understand the morphology of living tissues growing under constraints [42]. However, the role of creasing instabilities in contact mechanics has not been explored systematically due to their highly nonlinear features. In soft hydrogels, for example, the swelling ratios are significant such that the crease formations are difficult to control in experiments [43]. On the other hand, since PDMS elastomers comprise crosslinked networks swollen by a small amount of highly viscous solvents, the diffusion motion of long polymer chains in PDMS elastomers is often insignificant [44]. Therefore, creasing instabilities are not commonly considered for PDMS elastomers.

To explore the role of crease-like instabilities in soft contacts, we herein studied how swelling-induced surface patterns affect the adhesion energy of ultra-soft PDMS gels. The PDMS gels with elastic moduli of a few kPa can generate creasing instabilities across multiple length scales in a controlled fashion. We showed that the adhesion energy was greatly increased by the patterns even as the free chains in the gels were reduced significantly. Due to the broad use of PDMS in various engineering materials, our results will help the design of soft interfaces with enhanced adhesive properties.

## 2. Materials and Methods

### 2.1. Preparation of the Gel Substrates with the Instability Patterns

The soft PDMS gels were synthesized by pre-mixing the crosslinkers and polymer chains purchased from the Dow Corning CY52-276 (Dow Corning, Toray, Japan). The well-mixed solutions were filled in a Petri dish and then degassed for a few minutes until the trapped air bubbles were fully removed. The millimeter-thick samples were prepared in a Petri dish, whereas the micrometer-thick samples were spin-coated on a coverslip (Deckglaser, Marienfeld, Germany) at a speed of 800–3200 rpm. All the samples were left to cure at 40 °C for 24 h to obtain the crosslinked gel substrates for further characterizations. The resulting shear modulus (G) of the gels was around 1 kPa. We can estimate the average spacing between crosslinkers (a) by considering thermodynamic free energy of the polymer networks, which gives $G\sim k_{B}T/a^{3}$ [44]. For a given shear modulus of the soft gels, G ≈ 1 kPa, a is approximately in the order of 10 nm. To measure the precise thickness of the samples, the top and bottom interfaces of the gels were both coated with a layer of fluorescent beads. We located the focal planes of the two surfaces by using a fluorescent microscope equipped with a piezo-controlled sample stage (Olympus IX-73 Inverted Microscope, Tokyo, Japan), and the vertical displacement of the stage gave the sample thickness (see details in Appendix A). To generate the instability patterns, the cured substrates were mostly immersed in an excessive solution containing 100% toluene (99.9%, HPLC grade; Scharlau) for two days, and the solution was refreshed once every 24 h. During the swelling process, the samples were completely sealed to avoid any possible evaporation. After the swelling process was finished, the samples were further kept in a chemical hood for another 24 h to dry out the remaining toluene completely.

### 2.2. Optical Imaging of the Surface Profiles

To visualize the instability patterns distributed on the gel surfaces, we used an Olympus IX73 fluorescent microscope (Tokyo, Japan). The microscope can also work as an interferometer with a monochromatic filter (532/25 nm VIS Band Pass Filter) (Optolong Optics CO., Ltd., Kunming, China). To analyze the 3D morphology of the instabilities, we further carried out white-light interferometric measurements by using a surface profiler (Bruker NPFLEX, Billerica, MA, USA). The instrument could precisely reconstruct the 3D surface profiles and measure the average roughness and maximum vertical deformations of the substrates.

### 2.3. Adhesion Characterizations

The adhesion tests were performed on a texture analyzer (Mecmesin MultiTest 2.5, Mecmesin Ltd., West Sussex, UK) which was equipped with different shapes of indenters. To quantify the adhesion energy of each sample, we measured the force-displacement curves during quasistatic loading–unloading tests. In a typical experiment, the indenter approached the sample surfaces at a constant speed, 0.15 mm/min, and indented the substrates by roughly 10% of their thickness. The probe then slowly retracted to its initial height at the same speed after it was held at the maximum indentation for 1 min. Our indentation processes were quasistatic so that the viscoelastic dissipations from gels could be ignored in our measurements. The force was measured precisely by a force sensor (Mecmesin Ltd., West Sussex, UK) with a resolution of 0.4 mN. The experimental force-displacement results were compared with the classical Johnson-Kendall-Roberts theory. A Complementary Metal Oxide Semiconductor (CMOS) camera purchased from Thorlabs (Newton, NJ, USA) was also used to image the contact area during the indentations.

## 3. Results and Discussions

### 3.1. The Morphology of Surface Instabilities

Toluene solution greatly swelled the ultra-soft silicone gels that were prepared in the experiments. The swelling ratio in free space was found to be as large as 2.5. For gel samples fixed onto a glass substrate, the expansion restricted along the vertical direction inversely generated significant compressive strain along gel interfaces. After the toluene solution was removed, we observed clear instability patterns. It is worth noting that the large swelling ratio induced by toluene is essential for generating the surface patterns. For soft silicone gels immersed in ethanol or acetone solution, the substrates were swelled slightly, and no crease-like instabilities were observed as a result.

**Figure 1:**
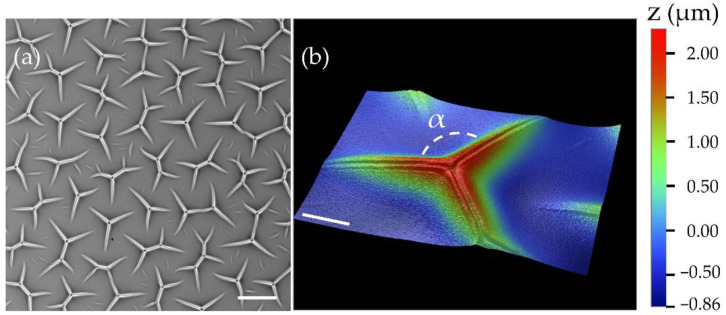
Representative microscope images of the gel substrates with surface instabilities. (**a**) A brightfield image (scale bar: 100 µm). (**b**) A 3D surface profile of an instability feature measured with a white-light interferometer (scale bar: 20 µm).

Figure 1a shows a representative microscope image of the surface of a 19-micrometer-thick gel substrate. The spatial distribution of patterns was largely uniform. Each individual feature comprised three arms with approximately equal lengths. When two individual features connected, they formed the joint patterns with four or five branches. A typical 3D profile of a feature with three branches measured by the surface profiler is shown in Figure 1b. The vertical height of the feature is slightly above 2 μm, and each arm is around 60 μm long. There are clear cuts at the center of the ridges, and the opening angle α between the two arms is close to 120°.

**Figure 2:**
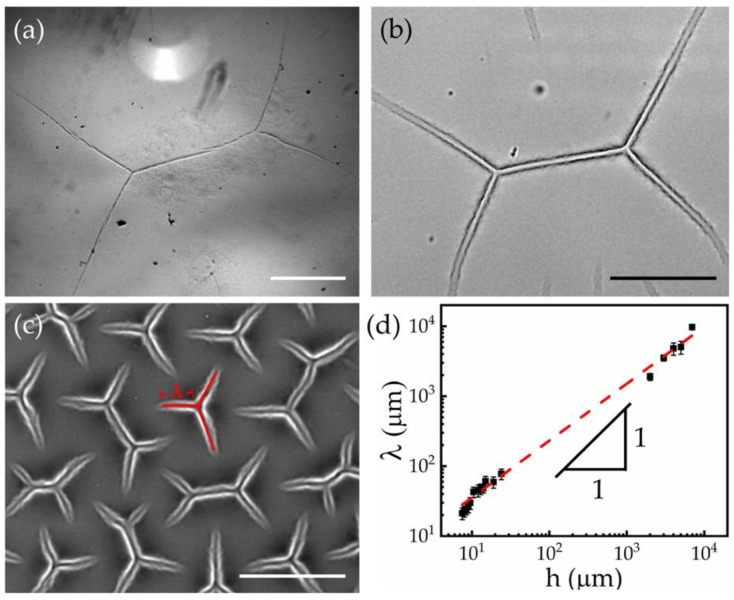
Instability features on samples with different thicknesses. Brightfield microscope images of the instability patterns generated on samples with the thicknesses of 1 mm (**a**), 24 µm (**b**), and 7.5 µm (**c**), respectively. The scale bars are 1 mm in (**a**) and 50 µm in (**b**,**c**). (**d**) Characteristic feature length (λ) is plotted against the sample thickness (h). The red dashed line indicates the best power-law fit to the experimental results, $\lambda =5.4\;h^{0.8}$.

Figure 2a–c shows the representative images of instability features as the sample thickness was varied from 1 mm to 7.5 µm. To observe the patterns across multiple length scales (as indicated by different scale bars in the panels (a) to (c)), the imaging measurements were performed by the objectives with different magnifications. The feature morphology remained universal across the scales, while their average dimensions decreased in accordance with the sample thickness (h). We systematically measured the feature sizes at the different sample thicknesses, and the results are summarized in Figure 2d. The characteristic arm length, λ, was defined as the average distance between the joint and the open ends of the branches (see Figure 2c). As the substrate thickness (h) increased from 7.5 μm to 7 mm, the resulting λ rose from 21.2 ± 4.1 μm to 9.7 ± 0.4 mm. By applying a power-law fit to the results in Figure 2d, we obtained an approximate scaling, $\lambda \sim h^{0.8}$, suggesting that the instability feature size was mainly determined by the sample thickness [32,45].

**Figure 3:**
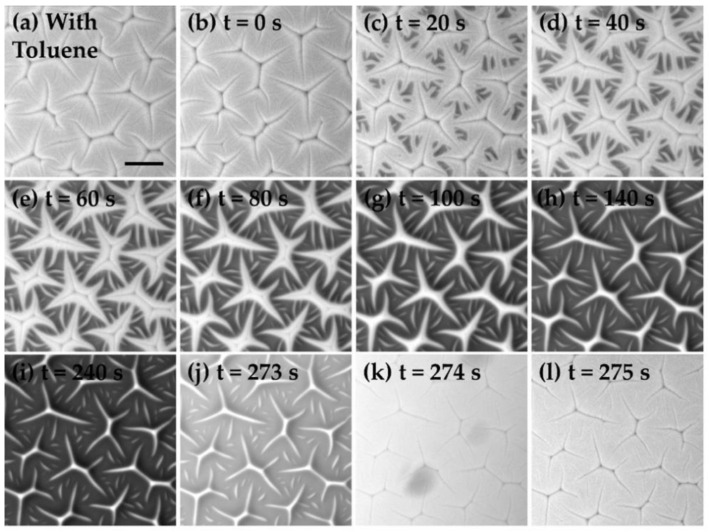
The growth and relaxation dynamics of the instability patterns on a 19-micrometer-thick substrate. (**a**) The sample was initially immersed in toluene. (**b**) At t=0, the toluene was removed with a pipette. (**c**–**i**) The relaxation of the gel surface during the drying process. (**j**) The moment when toluene was added again to the gel surface. (**k**,**l**) The gel surface swelled again in toluene, and the creasing instability reappeared. Scale bar in (**a**): 100 μm.

Notice that the surface profile shown in Figure 1b is not consistent with a typical creasing instability [18,42]. Creases normally appeared below the initial sample planes, while the features observed in our experiments comprised the ridges grown above the gel surfaces. To understand the underlying mechanism of these pattern formations, we imaged their growth and relaxation dynamics in situ under an optical microscope in Figure 3. A newly cured gel substrate was initially immersed in the toluene solution when we observed the creasing-like patterns as indicated by the cuts in the panel (a). At t=0 (Figure 3b), we quickly removed the toluene solution with a pipette so that the gel surface was exposed to air as the remaining toluene was evaporating. Figure 3c–i show how the surface morphology evolved during the drying process at t>0. At t=20 s, we began to observe islands of dark areas appearing between the creases, which were induced by the relaxation of the swollen surfaces. From the image series shown in the panels (c) to (i), the dark regions expanded their areas with time, and only the proximity of creases remained unmoved. As the drying of toluene continued, the surface profiles around the creases finally evolved into the ridges as seen in Figure 3i. The spatial positions and orientations of the resulting features aligned perfectly with the original creases. The finding also explains the cuts at the center of each instability branch in Figure 1b. As we re-immersed the substrate with the toluene solution at t=273 s (Figure 3j) again, the collapsed surfaces immediately re-expanded to their original states in less than a second (Figure 3k,l). The whole growth and relaxation processes shown in Figure 3 can be viewed in the Supplementary Video S1.

### 3.2. Adhesion Tests

**Figure 4:**
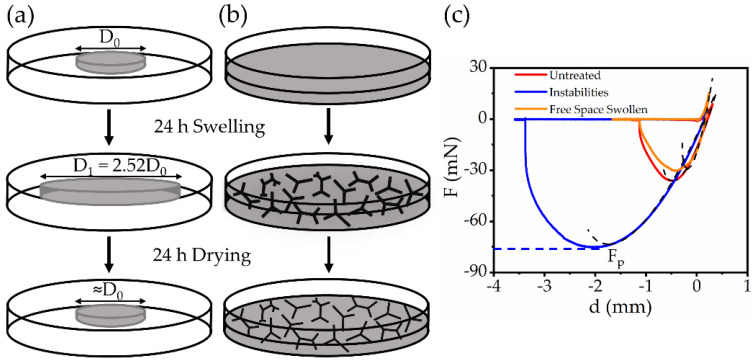
Adhesion tests on the gel substrates. (**a**) Schematics of the swelling process in free space. (**b**) Schematics of the swelling process in a confined Petri-dish. (**c**) The force–displacement curves in the indentation measurements for the untreated samples (red), the free swollen samples (orange), and the samples with instabilities (blue). The indenter used in the measurements was a steel spherical probe with a 16 mm diameter. The dashed black lines are the best fits of the JKR model to the experimental results.

We next explored the role of the instabilities in adhesive contacts. To quantify the changes of adhesion energy (W) due to the surface patterns, we prepared the soft gels through two different swelling protocols. First, the gel substrates were swollen in free space as illustrated by the schematics in Figure 4a. The swelling ratio was found to be approximately $D_{1}/D_{0}\approx 2.5$. After removing the toluene solution, the gels gradually retracted to their original sizes ($\sim D_{0}$). The sample surfaces remained smooth through this treatment in free space. By contrast, during the second preparation protocol, the swelling and drying processes were confined in a Petri-dish, and the resulting instability patterns appeared on the gel surfaces (see Figure 4b). We performed indentation tests on both samples and compared their results with the untreated gels. All the samples have a similar thickness of around 3 mm. To preserve the reproducibility of our results, all the measurements followed the standard loading–unloading approach discussed in Section 2.3. The force (F)—displacement (d) curves measured on the three soft interfaces were plotted with different colors in Figure 4c.

The force responses were measured by a spherical probe with a 16 mm diameter. We defined a pull-off force, $F_{p}$, by the force value recorded at the lowest point of the force–displacement curves in Figure 4c. The magnitude of $F_{p}$ determines the largest extension the gel surfaces can bear without detachment and indicates how sticky the substrates are. Here, we found that the samples with surface instabilities (blue line) bore a significantly larger pull-off force compared with the smooth samples (red and orange curves). We expected that the swelling of soft gels in both free and confined spaces would extract substantial free chains from the gels. According to previous studies [46,47], the absence of free polymer chains would potentially decrease the adhesion energy of an elastomer. Our experiments found that the substrates swollen in free space (orange) indeed exhibited a slight decrease (~30%) in the pull-off force compared with the untreated samples. By contrast, the instability patterns on the gel surfaces are the dominating factors of the adhesive responses as they increased the pull-off force, $F_{p}$, by a factor of greater than two. The details of the force measurements using the 6 mm diameter spherical probe and using the 16 mm diameter gel-coated probe (GC) are given in Appendix B.

We further quantitatively fitted the unloading force-displacement curves in Figure 4c to the Johnson-Kendall-Roberts (JKR) theory in contact mechanics [48]. In the JKR model, when a rigid spherical probe with a radius R is brought into contact with a soft substrate, the contact radius a induced by an external force F can be expressed as

(1) $$a^{3}=\left(9R/16E\right)\left[F+3\pi WR+\sqrt{6\pi RFW+{\left(3\pi WR\right)}^{2}}\;\right]$$

The adhesion energy (W) in Equation (1) represents the interfacial energy cost per unit area to separate two material surfaces. To evaluate the theoretical predictions of the force–displacement curves numerically, we considered a set of dimensionless parameters, $\hat{F}=F/\pi WR$, $\hat{d}=\beta^{2}d/R$ and $\hat{a}=\beta a/R$, where $\beta ={\left(4ER/3W\right)}^{1/3}$. As a result, the normalized force $\hat{F}$ and indentation depth $\hat{d}$ can be both expressed as functions of the normalized contact radius $\hat{a}$,

(2) $$\hat{F}=\left(4{\hat{a}}^{3}/3\pi \right)-\left(4{\hat{a}}^{\frac{3}{2}}/\sqrt{2\pi }\right)$$

(3) $$\hat{d}={\hat{a}}^{2}-\sqrt{2\pi \hat{a}}$$

By fitting Equations (2) and (3) numerically to the experimental results in Figure 4c, we could determine the adhesion energy (W) of each substrate. The dashed lines in Figure 4c indicate the best numerical fits to the experimental results. For the samples with the instabilities, the adhesion energy between the steel probe and substrates was found to be W=(2.2 ± 0.3) J/m^2^, which was more than twice the adhesion energy of the untreated samples, for which W=(1.0±0.1)  J/m^2^. By contrast, the adhesion energy of the free swollen substrates was slightly reduced to W=(0.8 ± 0.1)  J/m^2^. These results quantitatively confirm that the presence of the instability patterns plays a critical role in the adhesion enhancement.

We also obtained the effective Young’s modulus (E) of each sample from the fittings to the Hertz contact for the loading periods. However, it is important to mention that the standard contact models assume that the substrates are semi-infinitely thick. For a substrate with a finite thickness, the effective Young’s modulus (E) was overestimated due to a finite-size effect [49,50]. The true Young’s modulus of the substrates ($E_{0}$) needs to be corrected by $E_{0}=E\;\psi$ where ψ is a geometric constant determined by the sample thickness and probe radius. In our experiments, we calculated that ψ = 0.44 from the results of the finite element method [51]. Table 1 shows the pull-off force ($F_{p}$), adhesion energy (W), and the true Young’s modulus ($E_{0}$) for each sample in detail. Notice that the stiffness of free swollen samples was difficult to determine precisely since the swelling process deformed the network plastically. Here, we estimated that $E_{0}\approx 6$ kPa for the free swollen samples, which was about twice that of the untreated samples. This stiffening effect was caused by the collapse of the networks after removing the free chains.

### 3.3. Effects on the Contact Lines

**Figure 5:**
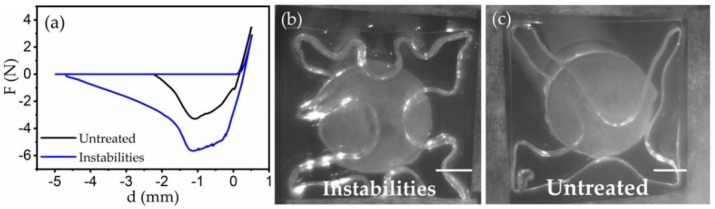
Contact line variations during the indentation tests. (**a**) Force-displacement curves for a glass coverslip indenting the samples with and without instabilities. (**b**) Fingering pattern for instabilities sample and (**c**) normal smooth contact line for untreated sample. Scale bar = 0.5 cm.

The instability-induced adhesion enhancement was associated with the variations of contact periphery in adhesion tests. To better visualize the contact lines between the gel and the probing surfaces, we attached a coverslip to a cylindrical probe. When the coverslip was in contact with different sample surfaces, we imaged the contact area from below while the probe was measuring the force response simultaneously. Figure 5a shows the comparison between the force curves of the samples with (blue) and without (black) instability patterns. As expected, the increase in adhesion energy was found again for gel surfaces with the instability patterns. Meanwhile, the contact lines between the coverslip and gel surfaces with instabilities (Figure 5b) became unstable compared with the untreated surfaces (Figure 5c). This difference can be clearly visualized in the Supplementary Video S2. Since the detachment at the contact interfaces involved significant surface deformations, increasing the contact perimeter could increase the energy cost to separate the interfaces between the probe and gels and therefore increase the effective adhesion energy [52]. To confirm the generality of this observation, we also imaged the contact area between the gels and spherical probes during the same indentation tests. For the samples with instabilities, we observed that the width of contact lines thickened (see the Supplementary Video S3). Combining these findings together, we conclude that the modifications of contact lines by the surface instabilities are essential for the adhesion enhancement. Further, the details of the similar tests conducted under different surface conditions are given in Appendix B.

**Figure 6:**
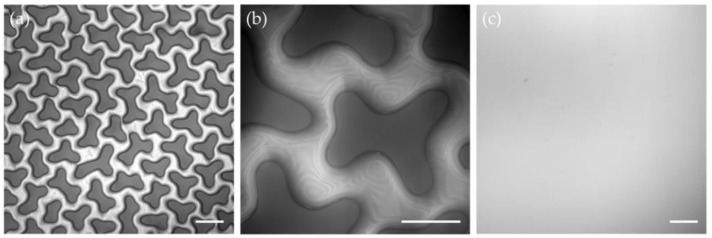
Microscope images of the contact areas. (**a**) A microscope image of the contact area when a glass coverslip was indenting a 19-micrometer-thick substrate with instability patterns. (**b**) Zoomed-in view of the contacts in (**a**) with an interference microscope. The interference fringes were induced by the trapped-air cavities. (**c**) A microscope image of contact area when a glass coverslip was indenting a 19-micrometer-thick substrate without the instability patterns. The scale bars in (**a**,**c**) are 100 µm. The scale bar in (**b**) is 50 μm.

We also evaluated the role of surface instabilities in soft contacts microscopically. To visualize the local details at the micrometer scale, we used an interference imaging setup by mounting a monochromatic filter cube (532/25 nm) to an optical microscope. We prepared microscale instability patterns on a 19 μm -thick substrate. A glass coverslip was then placed on the top and gradually pressed into the substrate. Due to the small refraction index mismatch between silicone gels and glasses, reflective light at the interfaces of the two materials was negligible. Therefore, the dark domains in Figure 6a represent the direct contacts between the gel and coverslip. From the geometric shapes of these contact domains, we conclude that they were the deformed instability features. The connected bright areas between these features were the trapped-air cavities. If we zoomed into these regimes, interface fringes from the air films were resolved by the interference microscope (see Figure 6b). By contrast, we also imaged the contact area between a glass coverslip and an untreated surface. As shown in Figure 6c, we did not observe any internal contact structures or interference fringes for the untreated surfaces, suggesting that there was no trapped air. Like the variations observed on contact periphery, we expect that these internal structures (Figure 6a,b) also greatly increased the total perimeter of contact lines and therefore enhanced the apparent adhesion energy.

## 4. Conclusions

In this work, we systematically studied how the swelling-induced instabilities on soft gels affect their adhesive properties. We demonstrated that permanent interfacial features could be generated on ultra-soft surfaces through creasing instabilities. The morphology of the features remained universal, and their sizes scaled almost linearly with the sample thickness over three orders of magnitude. We quantitatively showed that the instability patterns effectively increased the adhesion energy of the substrates under different measuring conditions. The adhesion enhancement could be related to the variations of contact lines both around and inside the contact area. We believe that these surface features are promising candidates for many engineering applications due to high compliance and adaptability. The results from the work will be helpful in the design of ultra-soft functional surfaces through other techniques, such as 3D printing or soft lithography. Compared with wrinkles, these patterns that emerge from the swelling of soft gels are better systems for modeling the formations of surface textures of various living tissues.

## Figures and Tables

**Table 1:** JKR analysis of the adhesion tests. The pull-off force ($F_{p}$), adhesion energy (W), and elastic modulus ($E_{0}$) for each soft substrate obtained from the indentation measurements in Figure 4c. The uncertainties were calculated from the standard deviations by repeating the measurements at least three times.

Samples	Pull-Off Force *F_p_* (mN)	Adhesion Energy *W* (J/m^2^)	Young’s Modulus *E*_0_ (kPa)
Untreated	38.6 ± 2.9	1.0 ± 0.1	2.6 ± 0.6
Instabilities	84.6 ± 12.3	2.2 ± 0.3	2.9 ± 0.4
Free space swollen samples	27.7 ± 10.2	0.8 ± 0.1	≈6.0

**Figure A1:**
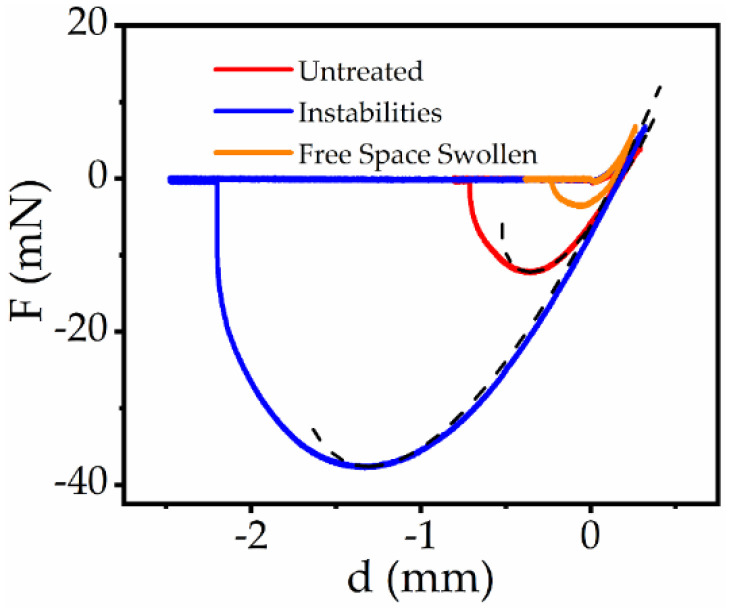
Adhesion tests performed with a 6 mm steel spherical probe. The red, orange, and blue curves represent the force-displacement curves measured for the untreated samples, the free swollen samples, and the samples with instabilities, respectively.

**Figure A2:**
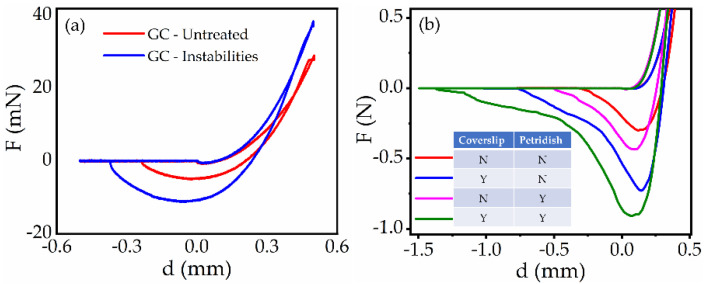
Adhesion tests between two silicone gel surfaces. (**a**) Force-displacement curves of the gel-coated spherical probe (16 mm) indenting gel substrates with (blue) and without instabilities (red). (**b**) Force-displacement curves of gel-coated coverslips indenting thick gel substrates. The experiments were performed under four different surface conditions. Inset table: Y indicates that the surface has instability patterns, and N indicates no instability.

## Data Availability

All data in the work are available upon request.

## Supplementary Materials

The following supporting information can be downloaded at: https://www.mdpi.com/article/10.3390/ma15030852/s1. Video S1: Growth and relaxation of surface instabilities, Video S2: Contact dynamics during adhesion detachment (with coverslips) for the samples with and without instabilities, Video S3: Variations in contact lines during adhesion detachment (with the 16 mm steel spherical probe) for the samples with and without instabilities.

## Appendix A

Table A1 shows the characteristic feature size (***λ***) for the different samples thicknesses (***h***) we have prepared varying from micrometer to millimeter scale.

**Table A1:** The characteristic feature size (***λ***) for different samples thicknesses (***h***).

Sample Thickness *h* (mm)	Feature Length *λ* (mm)	Sample Thickness *h* (µm)	Feature Length *λ* (µm)
2	1.9 ± 0.2	24	77.5 ± 13.2
3	3.5 ± 0.3	19	59.1 ± 10.9
4	4.8 ± 0.9	15	60.9 ± 10.5
5	5.0 ± 1.1	14	47.9 ± 6.5
7	9.7 ± 0.4	13	47.5 ± 8.2
		12	45.0 ± 8.8
		10.5	42.8 ± 7.3
		9.5	29.8 ± 5.8
		9	26.2 ± 4.6
		8.5	24.3 ± 4.2
		8	22.6 ± 3.8
		7.5	21.2 ± 4.1

## Appendix B

To confirm the generality of the conclusions that we drew in this work, we further quantified the role of surface instabilities in soft adhesion under different measuring conditions. For example, Figure A1 shows the force-displacement curves measured with a small steel spherical probe (diameter = 6 mm) on various interfaces. The overall trend was similar to the results obtained with the 16 mm spherical probe (Figure 4c). Additionally, besides the adhesive properties between gel surfaces and different testing materials, we investigated the contacts between the same materials. First, we coated the 16 mm spherical probe with a thin layer of soft silicone gels. When the probe was indenting the soft substrates, both surfaces were made of silicone gels. Figure A2a shows the force-displacement curves measured with this gel-coated probe (GC) on different sample surfaces. Second, we also used coverslips coated with soft silicone gels to indent another gel substrate. Figure A2b shows force-displacement curves measured under different surface conditions. In all these tests, we consistently found that instability patterns on gel surfaces substantially increased the adhesion energy.
